# New Insight in Vaccination and Public Health: A Commentary from Special Issue Editors

**DOI:** 10.3390/vaccines10020183

**Published:** 2022-01-25

**Authors:** Paolo Castiglia, Antonella Arghittu

**Affiliations:** 1 Department of Medical, Surgical and Experimental Sciences, University of Sassari, 07100 Sassari, Italy; 2 University Hospital of Sassari, 07100 Sassari, Italy

## 1. Background

Vaccination is a key component of primary health care and an indisputable human right [1,2]. Thanks to the success of vaccines, it has been possible to eradicate infectious diseases such as smallpox and drastically reduce the incidence of disabling diseases such as polio and measles [3,4].

The scientific evidence on the validity and safety of vaccines is widely described in the literature. In contrast, the historical weight of their importance, together with the real danger that infectious diseases pose to health, are significantly underestimated in common perception [5,6]. The COVID-19 pandemic has further highlighted the importance of vaccination as a powerful life-saving weapon in the fight against this infectious disease during the first waves following the introduction of the vaccine. In the current COVID-19 pandemic, in which the spread of the Omicron variant is putting a strain on the global health system, already burdened by the direct and indirect consequences of previous waves of the pandemic, there is still a discrepancy between the scientific evidence on the extraordinary effectiveness of vaccines in protecting against severe forms as well as lethality and the perception of the risk attributed to them [7].

In this regard, it is important to underline how an altered perception of health risk has a significant impact on the health decisions taken by the population. Indeed, according to the theory of ‘outrage’, coined by the American sociologist Peter Sandman, the emotional component surrounding the event plays a fundamental role in risk perception, and a discrepancy between the real danger and the perceived risk can lead to inappropriate behaviour that does not comply with the recommended public health measures [8,9,10].

In this regard, several studies describe how the decision-making process regarding health choices is strongly conditioned by what one reads online, in particular regarding vaccinations. As for most health issues, the issue of vaccination regularly faces the sudden demand for knowledge and shareability by users, which is the cause of the uncontrolled spread of sometimes false, mendacious and misleading information [9,10,11]. This, together with a latent climate of distrust in vaccination practices, has determined the negative drop in vaccination coverage that can be seen today in all population groups, with particular reference to the paediatric cohort [12].

This phenomenon, known as Vaccine Hesitancy, involves at least 15% of the general public and, even in the context of hospital care, has a negative influence on the perceptions, attitudes and behaviour of healthcare workers [13,14]. Scepticism towards vaccination on the part of healthcare workers is particularly evident for some vaccines, such as the flu vaccine, whose benefit in terms of reducing incidence, mortality and costs associated with the disease is widely underestimated even among healthcare workers. This is demonstrated by the discrepancy between the influenza vaccination coverage recommended by WHO (95%) and that commonly observed among health workers during the succession of flu seasons [6,15]. 

This is even more worrying if one considers that, due to the nature of their professional duty, healthcare workers have a two-fold responsibility towards their patients: (i) to guarantee the quality of the healthcare services they provide and (ii) to set good examples for the adoption of correct health behaviours. In this context, the study and implementation of strategies aimed at improving vaccination compliance within this category of professionals, as well as in the general public, through health education and continuous training activities are of utmost importance [15,16].

Nonetheless, in a landscape where the phenomenon of vaccination refusal, accompanied by a feeling of doubt and scepticism, has grown, the traditional tools of counteraction are proving ineffective [17,18]. Furthermore, during the COVID-19 pandemic, the continuous media exposure to an enormous volume of apparently conflicting news for an inexperienced user, such as the discrepancies in the impact of the disease in terms of the number of infections and mortality in different countries and in different time periods [19], as well as the conflicting opinions on the efficacy of the different vaccines available, made it difficult for people to find reliable and trustworthy sources of information [20]. This phenomenon, described by the World Health Organisation as ‘Infodemia’, is the critical response to an ‘identitary’ need by modern society, according to which the state of health is obtainable and defensible by the individual through independent access to current information technologies and sources dealing with psychophysical wellbeing and correct lifestyles. While this is identified as the best empowerment strategy to improve the ability of individuals to access and use information effectively, it also exposes the user to the immeasurable quantity of information of dubious truthfulness and to the various content found online [20,21,22].

Rapid and cheap access to the Internet and the large number of registered users on mobile applications (APPs) have made the Web an effective tool for spreading information; the social isolation imposed on the population has further contributed to the increased use of digital platforms. In view of this greater power of information sharing, Public Health must necessarily adapt by radically changing the way it communicates with users-patients [17,18]. 

In this perspective, bearing in mind the great potential offered by the Internet in the processes of seeking health information, the use of online channels by health institutions appears necessary to ensure the dissemination of medical-scientific knowledge among users and patients in order to support citizens in taking actions and making informed decisions regarding their own health. Thus, although there are now many approaches to encourage people to accept a vaccination (e.g., storytelling, celebrity endorsement, etc.), the use of institutional websites and alternative modes of communication promoted by scientific societies seem to have encouraging results in terms of improving user adherence to vaccination programmes [6,15,17,18]. 

In-depth analysis of these issues in a dedicated Special Issue, through a multidisciplinary overview, could be a useful tool for the scientific community to identify the best practices with proven effectiveness in the field for the implementation of decisive strategies aimed at improving population compliance and achieving the desired health objectives.

## 2. Conclusions

Based on these assumptions, the Special Issue “New Insight in Vaccination and Public Health”, published in the journal *Vaccines*, has the main aim of increasing the international literature evidence and observations in the field regarding (i) the adoption of vaccination strategies in order to improve the population’s vaccination coverage and (ii) tackling the phenomenon of Vaccine Hesitancy (VH) through communication and health education activities for both the general public and health professionals. 
